# Knowledge and practice of birth preparedness and complication readiness among pregnant women attending antenatal clinic in Openzinzi Hciii, Adjumani District, Uganda

**DOI:** 10.11604/pamj.2019.34.46.16869

**Published:** 2019-09-24

**Authors:** Masudio Florence, Catherine Atuhaire, Claude Ngwayu Nkfusai, Joyce Shirinde, Samuel Nambile Cumber

**Affiliations:** 1 Faculty of Medicine, Department of Nursing, Mbarara University of Science and Technology, Mbarara, Uganda; 2 Department of Microbiology and Parasitology, Faculty of Science, University of Buea, Buea, Cameroon; 3 Cameroon Baptist Convention Health Services (CBCHS), Yaoundé, Cameroon; 4 School of Health Systems and Public Health, Faculty of Health Sciences, University of Pretoria Private Bag X323, Gezina, Pretoria, 0001, South Africa; 5 Section for Epidemiology and Social Medicine, Department of Public Health, Institute of Medicine, The Sahlgrenska Academy at University of Gothenburg, Box 414, SE-405 Gothenburg, Sweden; 6 Faculty of Health Sciences, University of the Free State, Bloemfontein, South Africa

**Keywords:** Birth preparedness, complication readiness, pregnancy, antenatal, women, Adjumani

## Abstract

**Introduction:**

Every day, approximately 830 women globally die from pregnancy-child birth related complications and all maternal deaths are mainly due to the three phases of delay usually experienced in maternal care which originates from inadequate or lack of birth and emergency preparedness. Despite the benefit of Birth Preparedness and Complications Readiness (BPACR) in the reduction of the three phases of delay and thus reduction of maternal deaths and complications, no study has been conducted in Adjumani district to assess the knowledge and practice of birth preparedness and complication readiness, thus our objective was to assess the knowledge and practice of Birth Preparedness and Complications Readiness (BPACR) among pregnant women attending antenatal clinic at Openzinzi Health Centre (HC) III in Adjumani District.

**Methods:**

A descriptive cross sectional study design with a sample of 80 respondents was used for the study. Simple random sampling was used to select the respondents in the study area. A research administered questionnaire was used for data collection.

**Results:**

Most of the respondents (27.5%) were in the age group of 26-35 years. The majority 43.75% ended at primary level of education, 50% were unemployed, and the majority 71.25% and 70% knew identifying skilled birth attendants and health facilities respectively as components of BPACR. 76.25% of the respondents mentioned vaginal bleeding and 62.5% over vomiting as danger signs in pregnancy while 12.5% did not know any danger sign in pregnancy. 76.25% identified place for skilled birth, 66.25% identified skilled birth attendant, and only 15% identified blood donor.

**Conclusion:**

The practice of BPACR was poor among the pregnant women attending antenatal care at Openzinzi Health Centre III in Adjumani District. The knowledge about BPACR was higher among the educated respondents involved in the study.

## Introduction

Every day, approximately 830 women globally die from pregnancy-child birth related complications [1]. Ninety-nine percent of all maternal deaths occur in developing countries and more than half of these deaths occur in sub-Saharan Africa [2]. All maternal deaths are mainly due to the three phases of delay usually experienced in maternal care and this originates from inadequate or lack of birth and emergency preparedness [3]. Birth preparedness helps ensure that women can reach professional delivery care when labor begins. This also reduces the delays that occur when women experience obstetric complications since it ensures the readiness and timely utilization of skilled maternal and neonatal health care [4]. Birth Preparedness and Complication Readiness (BPACR) is a strategy to promote the timely use of skilled maternal and neonatal care, especially during childbirth and will ensure that women can have professional delivery thus reducing obstetric complications [5]. The major components of BPACR involved knowledge of danger signs, identification of a skilled birth attendant; identification of the closest appropriate care facility, plan for transportation to this care facility for delivery and/or obstetric emergencies, save money to pay for care and other resources, identification of a potential blood donor and decision maker in case of emergency [6]. Women die as a result of complications during pregnancy.

Most maternal deaths are preventable, as the health-care solutions to prevent or manage complications are well known [7]. All women need access to antenatal care in pregnancy, skilled care during child birth, and care and support in the weeks after child birth. The high number of maternal deaths in some areas of the world reflects inequities in access to these health services [8]. Knowledge of pregnant mothers on BPACR improves recognition of any problem in pregnancy, reduces the delay in deciding to seek care and provides information on appropriate sources of care making the care-seeking process more efficient [9]. Pregnant women, their families, and communities are encouraged to effectively plan for births and deal with any anticipated emergencies, if they occur [6]. Timely management and treatment by skilled health professionals of a pregnant mother can make the difference between life and death for both the mother and the baby. Unfortunately, poor women in remote areas are the least likely to receive adequate health care [7]. In spite of the benefit of BPACR in the reduction of the three phases of delay and thus reduction of maternal as well as neonatal deaths and complications [10], no study has been documented that assessed the knowledge and practice of birth preparedness and complication readiness in Adjumani, Uganda. The study assessed the knowledge and practice of BPACR among pregnant mothers attending antenatal clinic in Openzinzi Health Centre III in Adjumani district, Uganda.

## Methods

**Study setting:** the study design was cross-sectional in nature, employing quantitative methods of data collection. It was conducted in Openzinzi HC III in Adjumani district which is 5km away from Adjumani town where the district general hospital is located. The study focused on the outpatient department especially antenatal care section. The health centre is located in Adropi sub-county in Adjumani district. Adjumani district is located in the northern part of Uganda in the West Nile sub region and it is approximately 444.9km from Kampala, the capital city of Uganda.

**Population:** the study targeted pregnant mothers from the age of 15 years to 45 years attending antenatal clinic in the study area.

**Sample size:** Yamane (1967) provides a simplified formula to calculate sample size. This formula was used to calculate the sample sizes. A 95% confidence level and P= 0.05 are assumed for the equation below.

$$N=\frac{n}{1+N{\left(e\right)}^{2}}$$

N= Total number of nurses from units that were considered estimated to 47 nurses (hospital's personnel office October 2015); n= desired sample size; e= desired level of precision at 5%, with a 95% confidence interval. When the above equation is applied to the sample of nurses used, N= 96/1+96 (0.05)^2^ =80.

**Sampling technique:** this study employed simple random sampling method to recruit participants in the study. The researcher identified the pregnant mothers attending antenatal care clinic in the study area who were willing to participate in the study. The researcher then made papers on which she manually wrote two separate papers with yes and no respectively. These were folded and placed in a box and then study participants were told to pick a paper from the box once without returning it back into the box and one who picked paper with a yes was allowed to participate in the study. This was done on the daily basis until the required number was reached.

**Data collection tools and administration of questionnaires:** research instrument used was a questionnaire. The designed questionnaire was translated into the local language and back translated. The research instrument had 17 questions and took approximately 15 minutes to administer it. The instrument adapted from questionnaire formulated by Mohammed [11] was formulated to obtain data in relation to the study objectives. All participants who consented were interviewed using a structured questionnaire. Prior to its use in this study, a total of 10 questionnaires were pretested at the Mbarara University Teaching Hospital among pregnant women attending antenatal care (ANC) with the aim of revising poorly structured questions, estimate the average time required to fill the questionnaire and thus validate the use of the questionnaire in our context. Knowledge on BPACR consisted of 12 questions and each correct response was scored as 1 and 0 for a wrong response. The knowledge scores for an individual was calculated and summed up to give a total knowledge score on 12. A score between 0-4 was classified as poor, 4-8 as good and 9-12 as excellent adapted from a study conducted by Abongwa *et al.* [12]. The research questions investigated were: knowledge on components of BPACR: identifying means of transport; identifying health facility; identifying skilled birth attendant; identifying blood donor. Knowledge on danger signs during pregnancy: vaginal bleeding; over vomiting; oedema, Headache. Practice of BPACR: saved money; identifying means of transportation; identifying place of skilled birth; identifying skilled birth attendant; identifying blood donor.

**Data collection procedure:** prior to carrying out the study, a pre-visit to the study area was made to seek permission and to familiarize with the daily work routines so as to get the best time for the data collection exercise. Data was generated using questionnaires and informed consents were obtained from respondents before the interview. At the end of the questionnaire administration, participants were encouraged to ask questions which were answered to participants satisfaction.

**Data management:** data was entered into excel. Descriptive analysis was used and data presented in form of tables, graphs and pie-charts.

**Implication to practice:** increasing awareness about birth preparedness and complications readiness components and encouraging the practice among pregnant women is an important role and responsibility within the nursing profession. Nurses can make a significant difference with continuous health education, involvement of the community members, other stake holders to boost the knowledge and increase the practice of birth preparedness and complication readiness among the pregnant mothers and the community at large.

**Implication to policy:** the result of poor practice of birth preparedness and complication readiness among the respondents is a very significant issue in the nursing profession and nurses can use this as a guide to develop policies and support systems to empower the pregnant women to improve the practice of birth preparedness and complications readiness in the study area and the country at large.

**Ethical considerations:** once the department of nursing approved the study, an introductory letter from the Head of the Nursing Department was attached on submission to the Faculty Research Committee (FRC) and then to Research Ethics Committee (REC). The ethical approval for the study was obtained from the REC of Mbarara University of Science and Technology. We obtained approval and permission to conduct the study from the director of Openzinzi Health Centre III. Participation was voluntary and informed written consent was provided by each participant. The right to decline or to withdraw from the study at any stage without incurring any penalty was explained. All data were kept locked up and accessible only to the researcher.

**Limitations of the study:** the study only involved pregnant mothers from 15 years to 45 years and yet there may be some mothers below 15 years who may be pregnant mothers attending antenatal clinic at the study area. The study population therefore might not be a representative of all the pregnant mothers attending antenatal care clinic at the study area.

## Results

**Respondents demographic characteristics:** from the results, 45% of participants had at least attended antenatal care once and 38.75% were pregnant for their first time. 27.5% (n=22) were between the age group of 26-35 years, most of them 80% (n=64) were married. The majority 43.75% (n=35) ended at primary level education, half of them 50% (n=80) were unemployed, 38.75% (n=31) were carrying their first pregnancy and the majority attended antenatal care service only once with the current pregnancy (Table 1).

**Table 1:** Respondents’ demographic characteristics

Variables	Frequency	Percentage (%)
**Number of pregnancy**		
1^st^	31	38.75
2^nd^	20	25
3^rd^	6	7.5
4^th^	6	7.5
5^th^	13	16.25
6^th^	3	3.75
7^th^	1	1.25
**Number of antenatal care visits**		
Once	36	45
Twice	28	35
Thrice	10	12.5
Quadruple	6	7.5
**Age(years)**		
15-17	17	21.25
18-25	20	25
26-35	22	27.5
36-45	21	26.25
**Marital status**		
Single	12	15
Married	64	80
Divorced	4	5
**Education level**		
Primary	35	43.75
Secondary	9	11.25
Tertiary	19	23.75
not attended	17	21.25
**Occupation**		
Student	5	6.25
self employed	20	25
civil servant	15	18.75
Unemployed	40	50
**Religion**		
Catholic	46	57.5
Protestants	23	28.75
Muslims	11	13.75

**Figure 1:**
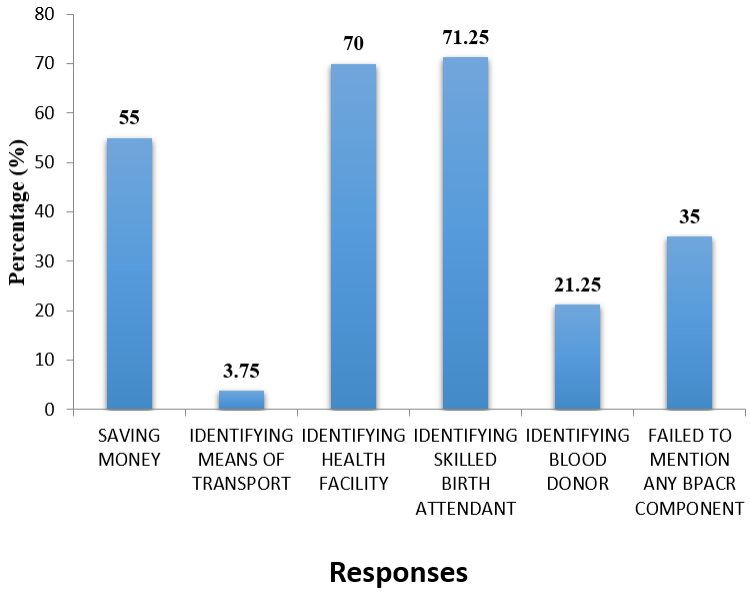
Knowledge on components of BPACR

**Knowledge on danger signs during pregnancy:** 71.25% and 70% of the respondents mentioned identifying skilled birth attendant and identifying a health facility respectively as the components of BPACR (Figure 1).

**Practice of BPACR among the respondents:** 76.25% of the respondents mentioned vaginal bleeding and 62.5% over vomiting as danger signs in pregnancy while 12.5% did not know any danger sign in pregnancy (Table 2).

**Table 2:** Knowledge on danger signs during pregnancy

Age Group (Years)	VaginalBleeding	OverVomiting	Oedema	Headache	No Knowledge
15-17	13	7	1	1	2
18-25	13	11	1	0	2
26-35	16	14	11	6	4
36-45	19	18	12	7	2
Total	61	50	25	14	10
**Percentage**	**76.25**	**62.5**	**31.25**	**17.5**	**12.5**

**Knowledge on components of BPACR:** 76.25% identified place for skilled birth, 66.25% identified skilled birth attendant, and only 15% identified blood donor (Table 3).

**Table 3:** Practice of BPACR among the respondents

Age (years)	Saved money	Identified transport means	Identified place for skilled birth	Identified skilled birth attendant	Identified blood donor
15-17	0	7	12	8	0
18-25	2	10	18	16	0
26-35	11	18	17	17	8
36-45	8	11	14	12	4
Total	21	46	61	53	12
**Percentage**	**26.25**	**57.5**	**76.25**	**66.25**	**15**

## Discussion

The age groups of the respondents were evenly distributed. This is probably due to the fact that, most of the women in the area are in their reproductive age and are sexually active thus had almost equal chances of becoming pregnant, specifically the age group of 26-35 years, being the ages for marriage for many women and hence increased rate of pregnancies. The relatively high number of teenage pregnancy is a concern in this study since the risk of maternal death is reported high among the teenagers [7] and this is evidenced by the statistical analysis of the study whereby, out of the 17 pregnant mothers in the age group of 15-17years, 82.35% did not know any component of BPACR and only 18.75% of them had knowledge about the danger signs during pregnancy, this probably may be one of the reasons why there is high maternal death among the teenagers. About the number of pregnancy, the majority of the respondents were pregnant for the first time (38.75%). The high percentage of those who are pregnant for the first time is attributed to the slightly high number of pregnant teenagers of which out of 17 pregnant teenagers, 12(70.59%) of them were pregnant for the first time. Being pregnant for the first time is linked with poor knowledge and practice of BPACR. This is probably because they may be lacking the experience of how to prepare for birth and complication.

Regarding the education level, most of the respondents (43.75%) ended at primary level education. The study being in a rural area of Adjumani district, the level of girl child education may still be low coupled with early marriage in the rural areas. In the study, knowledge on BPACR was seen to be higher among the educated respondents especially those who attained secondary and tertiary level of education than those who did not attend any formal education and those who ended in primary education, this corresponds to the study finding of [13] who found out that knowledge of BPACR was higher among the educated than the uneducated ones. This is probably because the level of knowledge increases as one goes higher with education and also becomes more aware about health related issues.

In relation to the occupation of the respondents, half of the respondents (50%) were unemployed. This is probably because of the low levels of education attained by most of the respondents. Unemployment is one key factor which affects maternal health in Uganda since it is also found out that one of the barriers to accessing maternal health care services in Uganda includes financial limitations [14]. On the marital status majority of the respondents (80%) were married. This is because in African tradition being married is associated with child bearing hence higher rates of pregnancy among the married. On the religion, majority of the respondents were Catholics (57.5%). This is probably because majority of the people in the area are Catholics hence represents a greater percentage in antenatal care attendance thus the majority in the study. Regarding the number of antenatal attendance with the current pregnancy, most of the respondents (45%) have attended antenatal care only once. This may be so because most of the respondents are carrying their first pregnancy and may not be aware of the required number and frequency of antenatal attendance. Attending antenatal care is very significant because most of the pieces of information about pregnancy and child birth for example about BPACR are given during antenatal care services [4]. In assessing the knowledge of the respondents about the components of BPACR, majority of the respondents 71.25% and 70% had knowledge about identifying skilled birth attendant and health facility. None of the respondents mentioned having knowledge of danger signs during pregnancy as a component of BPACR, this corresponds to a similar study done by [15] where majority (86.2%) of their respondents knew that there is need to identify health care facility for delivery and also another study found out that 65% of the pregnant women were aware that there is need to save money and identify a delivery place [16].

High level of knowledge about identifying health facility for birth can be attributed to the fact that pregnant mothers are always informed to identify place of birth mostly in the first visit of antenatal making them to be more aware and informed about it as a component of BPACR. Identifying a health facility for birth means that the pregnant mothers will be assisted by a skilled birth attendant at the health facility and hence greater percentage of the respondents had knowledge about identifying a skilled birth attendant. Although most of the respondents knew that there is need to identify health facility and skilled birth attendants, only a few of the respondents (3.75%) knew that they should identify means of transport. It is very important that pregnant women should be knowledgeable about all the components of BPACR since high knowledge about components of BPACR ultimately empowers pregnant women and their families to make prompt decisions to seek care from skilled birth attendants [10]. Regarding knowledge about danger signs during pregnancy, most of the respondents (76.25%) knew vaginal bleeding during pregnancy and 62.5% knew over vomiting as danger signs in pregnancy (Table 2). Only 12.5% did not know any danger sign in pregnancy. In a similar study done in India by Acharya *et al.* [17] it was however found that awareness regarding danger signs of pregnancy was 27.8%, and majority of the women did not know any danger signs in pregnancy. Also, [14] stated that only 14.8% of the women knew obstetric danger signs in pregnancy and obstetric danger signs most commonly known included vaginal bleeding during pregnancy. Most of the respondents in this study were aware about vaginal bleeding as a danger sign in pregnancy because most pregnant women originally know that no vaginal bleeding is expected during pregnancy and any unexplained vaginal bleeding during pregnancy may be indicating a complication or dangers. On assessing the practice of the respondents regarding BPACR, most of the respondents (76.25%) identified place for skilled birth and 66.25% identified skilled birth attendant (Table 3). Identification of place for skilled birth and identification of skilled birth attendant are highly practiced components of BPACR components probably because of the high knowledge of the respondents about identifying birth attendant and health facility as components of BPACR.

This study’s finding corresponds to a study done in Nigeria by Tobin *et al.* [18], where 87.4% had identified a skilled care. However this finding is not in line with a similar study done by Gebre *et al.* [19] among 578 pregnant women where only 10.7% of pregnant women identified skilled provider, 18.1% arranged transportation to the health facility and 43.6% identified health facility for delivery and/or for obstetric emergencies. Although the study respondents had poor knowledge about identifying transport as a component of BPACR with only 3.75% having the knowledge, 46% of the respondents had identified means of transport for emergency, however the most commonly mentioned means of transport by the respondents is use of a bicycle (54.75%) which is owned by the majority of the households in the area. This is not an impressive result since it is well known that bicycles cannot help a lot in case of obstetric emergencies coupled to its speed, longer distance and poor geographic locations of some of the places in the study area. Only 26.25% of the respondents had saved money for child birth and emergency most likely because majority of the respondents were unemployed and hence may be lacking direct source of income which can enable them save for emergencies, a study in Nigeria [18] also showed that only 11.3% had saved money for obstetric care. Basing on the fact that majority of the respondents knew vaginal bleeding as obstetric danger signs, only 15% identified blood donor in case of emergencies, this makes it challenging to save the lives of the mothers in case of severe vaginal bleeding coupled with scarcity of blood in the health facility.

## Conclusion

Although the study found out that the respondents had knowledge about BPACR, the practice of BPACR was poor among the pregnant mothers attending antenatal care at Openzinzi Health Centre III in Adjumani district. The knowledge about BPACR was higher among the educated respondents involved in the study. The respondents had least knowledge about identifying transport as a component of BPACR.

### What is known about this topic

- All maternal deaths are mainly due to the three phases of delay usually experienced in maternal care which originates from inadequate or lack of birth and emergency preparedness;
- Knowledge of pregnant mothers on BPACR improves recognition of any problem in pregnancy, and reduces the delay in deciding to seek care;
- Birth Preparedness and Complication Readiness (BPACR) is a strategy to promote the timely use of skilled maternal and neonatal care.

### What this study adds

- Pregnant women in this study had good knowledge on BPACR but poor practice of BPACR;
- Identification of means of transport as a component of BPACR was the least known by the respondents;
- Knowledge of BPACR was poor among the pregnant teenagers.

## Competing interests

The authors declare no competing interests.
